# Researching COVID to enhance recovery (RECOVER) pediatric study protocol: Rationale, objectives and design

**DOI:** 10.1101/2023.04.27.23289228

**Published:** 2023-05-12

**Authors:** Rachel Gross, Tanayott Thaweethai, Erika B. Rosenzweig, James Chan, Lori B. Chibnik, Mine S. Cicek, Amy J. Elliott, Valerie J. Flaherman, Andrea S. Foulkes, Margot Gage Witvliet, Richard Gallagher, Maria Laura Gennaro, Terry L. Jernigan, Elizabeth W. Karlson, Stuart D. Katz, Patricia A. Kinser, Lawrence C. Kleinman, Michelle F. Lamendola-Essel, Joshua D. Milner, Sindhu Mohandas, Praveen C. Mudumbi, Jane W. Newburger, Kyung E. Rhee, Amy L. Salisbury, Jessica N. Snowden, Cheryl R. Stein, Melissa S. Stockwell, Kelan G. Tantisira, Moriah E. Thomason, Dongngan T. Truong, David Warburton, John C. Wood, Shifa Ahmed, Almary Akerlundh, Akram N. Alshawabkeh, Brett R. Anderson, Judy L. Aschner, Andrew M. Atz, Robin L. Aupperle, Fiona C. Baker, Venkataraman Balaraman, Dithi Banerjee, Deanna M. Barch, Arielle Baskin-Sommers, Sultana Bhuiyan, Marie-Abele C. Bind, Amanda L. Bogie, Natalie C. Buchbinder, Elliott Bueler, Hülya Bükülmez, B.J. Casey, Linda Chang, Duncan B. Clark, Rebecca G. Clifton, Katharine N. Clouser, Lesley Cottrell, Kelly Cowan, Viren D’Sa, Mirella Dapretto, Soham Dasgupta, Walter Dehority, Kirsten B. Dummer, Matthew D. Elias, Shari Esquenazi-Karonika, Danielle N. Evans, E. Vincent S. Faustino, Alexander G. Fiks, Daniel Forsha, John J. Foxe, Naomi P. Friedman, Greta Fry, Sunanda Gaur, Dylan G. Gee, Kevin M. Gray, Ashraf S. Harahsheh, Andrew C. Heath, Mary M. Heitzeg, Christina M. Hester, Sophia Hill, Laura Hobart-Porter, Travis K.F. Hong, Carol R. Horowitz, Daniel S. Hsia, Matthew Huentelman, Kathy D. Hummel, William G. Iacono, Katherine Irby, Joanna Jacobus, Vanessa L. Jacoby, Pei-Ni Jone, David C. Kaelber, Tyler J. Kasmarcak, Matthew J. Kluko, Jessica S. Kosut, Angela R. Laird, Jeremy Landeo-Gutierrez, Sean M. Lang, Christine L. Larson, Peter Paul C. Lim, Krista M. Lisdahl, Brian W. McCrindle, Russell J. McCulloh, Alan L. Mendelsohn, Torri D. Metz, Lerraughn M. Morgan, Eva M. Müller-Oehring, Erica R. Nahin, Michael C. Neale, Manette Ness-Cochinwala, Sheila M. Nolan, Carlos R. Oliveira, Matthew E. Oster, R. Mark Payne, Hengameh Raissy, Isabelle G. Randall, Suchitra Rao, Harrison T. Reeder, Johana M. Rosas, Mark W. Russell, Arash A. Sabati, Yamuna Sanil, Alice I. Sato, Michael S. Schechter, Rangaraj Selvarangan, Divya Shakti, Kavita Sharma, Lindsay M. Squeglia, Michelle D. Stevenson, Jacqueline Szmuszkovicz, Maria M. Talavera-Barber, Ronald J. Teufel, Deepika Thacker, Mmekom M. Udosen, Megan R. Warner, Sara E. Watson, Alan Werzberger, Jordan C. Weyer, Marion J. Wood, H. Shonna Yin, William T. Zempsky, Emily Zimmerman, Benard P. Dreyer

**Affiliations:** 1 Department of Pediatrics, New York University Grossman School of Medicine, New York, NY, USA.; 2 Department of Biostatistics, Massachusetts General Hospital, Boston, MA, USA.; 3 Department of Pediatrics, Columbia University Vagelos College of Physicians and Surgeons, New York, NY, USA.; 4 Department of Laboratory Medicine and Pathology, Mayo Clinic Hospital, Rochester, MN, USA.; 5 Avera Research Institute, Avera Health, Sioux Falls, SD, USA.; 6 Department of Pediatrics, University of California San Francisco, San Francisco, CA, USA.; 7 Department of Sociology, Lamar University, Beaumont, TX, USA.; 8 Department of Child and Adolescent Psychiatry, New York University Grossman School of Medicine, New York, NY, USA.; 9 Public Health Research Institute and Department of Medicine, Rutgers New Jersey Medical School, Newark, NJ, USA.; 10 Center for Human Development, Cognitive Science, Psychiatry, Radiology, University of California San Diego, La Jolla, CA, USA.; 11 Department of Medicine, Harvard Medical School, Boston, MA, USA.; 12 Department of Medicine, New York University Grossman School of Medicine, New York, NY, USA.; 13 Department of Physical Medicine and Rehabilitation, Virginia Commonwealth University School of Nursing, Richmond, VA, USA.; 14 Department of Pediatrics, Division of Population Health, Quality, and Implementation Sciences (POPQuIS), Rutgers Robert Wood Johnson Medical School, New Brunswick, NJ, USA.; 15 Department of Pediatrics, Columbia University Medical Center: Columbia University Irving Medical Center, New York, NY, USA.; 16 Department of Infectious Diseases, Children’s Hospital Los Angeles and the Keck School of Medicine, University of Southern California, Los Angeles, CA, USA.; 17 Department of Population Health, New York University Grossman School of Medicine, New York, NY, USA.; 18 Department of Cardiology, Boston Children’s Hospital, Boston, MA, USA.; 19 Department of Pediatrics, University of California San Diego School of Medicine, San Diego, CA, USA.; 20 School of Nursing, Virginia Commonwealth University, Richmond, VA, USA.; 21 Departments of Pediatrics and Biostatistics, University of Arkansas for Medical Sciences, Little Rock, AR, USA.; 22 Department of Child and Adolescent Psychiatry, Hassenfeld Children’s Hospital at NYU Langone, New York, NY, USA.; 23 Department of Pediatrics, Division of Child and Adolescent Health, Columbia University Vagelos College of Physicians and Surgeons and NewYork-Presbyterian, New York, NY, USA.; 24 Division of Pediatric Respiratory Medicine, University of California San Diego, San Diego, CA, USA.; 25 Division of Pediatric Cardiology, University of Utah and Primary Children’s Hospital, Salt Lake City, UT, USA.; 26 Department of Pediatrics, Children’s Hospital Los Angeles, Los Angeles, CA, USA.; 27 Department of Pediatrics and Radiology, Children’s Hospital Los Angeles, Los Angeles, CA, USA.; 28 Department of Pulmonary Research, Rady Children’s Hospital-San Diego, San Diego, CA, USA.; 29 College of Engineering, Northeastern University, Boston, MA, USA.; 30 Division of Pediatric Cardiology, NewYork-Presbyterian/Columbia University Irving Medical Center, New York, NY, USA.; 31 Department of Pediatrics, Hackensack University Medical Center, Hackensack, NJ, USA.; 32 Department of Pediatrics, Medical University of South Carolina, Charleston, SC, USA.; 33 Oxley College of Health Sciences, Laureate Institute for Brain Research, Tulsa, OK, USA.; 34 Center for Health Sciences, SRI International, Menlo Park, CA, USA.; 35 Department of Pediatrics, Kapiolani Medical Center for Women and Children, Honolulu, HI, USA.; 36 Department of Pathology and Laboratory Medicine, Children’s Mercy Hospital, Kansas City, MO, USA.; 37 Department of Psychological & Brain Sciences, Psychiatry, and Radiology, Washington University in St. Louis, Saint Louis, MO, USA.; 38 Department of Psychology, Yale University, New Haven, CT, USA.; 39 Department of Pediatrics, University of Oklahoma Health Science Center, Oklahoma City, OK, USA.; 40 Center for Human Development, University of California San Diego, San Diego, CA, USA.; 41 Department of Pediatrics, Division of Rheumatology, The MetroHealth System, Case Western Reserve University, Cleveland, OH, USA.; 42 Department of Neuroscience and Behavior, Barnard College - Columbia University, New York, NY, USA.; 43 Department of Diagnostic Radiology and Nuclear Medicine, University of Maryland School of Medicine, Baltimore, MD, USA.; 44 Departments of Psychiatry and Pharmaceutical Sciences, University of Pittsburgh, Pittsburgh, PA, USA.; 45 Biostatistics Center, George Washington University, Washington, DC, USA.; 46 Department of Pediatrics, Hackensack Meridian School of Medicine, Nutley, NJ, USA.; 47 Department of Pediatrics, West Virginia University, Morgantown, WV, USA.; 48 Department of Pediatrics, Robert Larner M.D. College of Medicine at the University of Vermont, Burlington, VT, USA.; 49 Department of Pediatrics, Rhode Island Hospital, Providence, RI, USA.; 50 Department of Psychiatry and Biobehavioral Sciences, University of California Los Angeles, Los Angeles, CA, USA.; 51 Department of Pediatrics, Norton Children’s Hospital, University of Louisville, Louisville, KY, USA.; 52 Department of Pediatrics, Division of Infectious Diseases, University of New Mexico, Albuquerque, NM, USA.; 53 Department of Pediatrics, University of California San Diego, San Diego, CA, USA.; 54 Division of Cardiology, Children’s Hospital of Philadelphia, Philadelphia, PA, USA.; 55 Arkansas Children’s Research Institute, Arkansas Children’s Hospital, Little Rock, AR, USA.; 56 Department of Pediatrics, Yale School of Medicine, New Haven, CT, USA.; 57 Department of Pediatrics, Children’s Hospital of Philadelphia, Philadelphia, PA, USA.; 58 Department of Cardiology, Children’s Mercy Kansas City, Ward Family Heart Center, Kansas City, MO, USA, Kansas City, MO, USA.; 59 Department of Neuroscience, University of Rochester School of Medicine and Dentistry, Rochester, NY, USA.; 60 Institute for Behavioral Genetics and Department of Psychology and Neuroscience, University of Colorado Boulder, Bolder, CO, USA.; 61 Pennington Biomedical Research Center Clinic, Pennington Biomedical Research Center, Baton Rouge, LA, USA.; 62 Department of Pediatrics, Rutgers Robert Wood Johnson Medical School, New Brunswick, NJ, USA.; 63 Department of Psychiatry and Behavioral Sciences, Medical University of South Carolina, Charleston, SC, USA.; 64 Department of Pediatrics, Division of Cardiology, George Washington University School of Medicine & Health Sciences, Washington, DC, USA.; 65 Department of Psychiatry, Washington University School of Medicine, St Louis, MO, USA.; 66 Department of Psychiatry, University of Michigan, Ann Arbor, MI, USA.; 67 Division of Practice-Based Research, Innovation, & Evaluation, American Academy of Family Physicians, Leawood, KS, USA.; 68 Departments of Pediatrics and Physical Medicine & Rehabilitation, Section of Pediatric Rehabilitation, University of Arkansas for Medical Sciences, Little Rock, AR, USA.; 69 Center for Health Equity and Community Engaged Research and Department of Population Health Science and Policy, New York, NY, USA.; 70 Clinical Trials Unit, Pennington Biomedical Research Center, Baton Rouge, LA, USA.; 71 Division of Neurogenomics, Translational Genomics Research Institute, Phoenix, AZ, USA.; 72 Department of Pediatrics, University of Arkansas for Medical Sciences, Little Rock, AR, USA.; 73 Department of Psychology, University of Minnesota, Minneapolis, MN, USA.; 74 Department of Pediatrics, Arkansas Children’s Hospital, University of Arkansas Medical School, Little Rock, AR, USA.; 75 Department of Psychiatry, University of California San Diego, La Jolla, CA, USA.; 76 Department of Obstetrics, Gynecology, and Reproductive Sciences, University of California San Francisco, San Francisco, CA, USA.; 77 Department of Pediatrics, Pediatric Cardiology, Lurie Children’s Hospital, Northwestern University Feinberg School of Medicine, Chicago, IL, USA.; 78 Departments of Pediatrics, Internal Medicine, and Population and Quantitative Health Sciences, Case Western Reserve University, Cleveland, OH, USA.; 79 Department of Pediatric Clinical Research, Medical University of South Carolina, Charleston, SC, USA.; 80 Department of Physics, Florida International University, Miami, FL, USA.; 81 Department of Pediatrics, Respiratory Medicine Division, University of California San Diego, San Diego, CA, USA.; 82 Heart Institute, Cincinnati Children’s Hospital Medical Center, Cincinnati, OH, USA.; 83 Department of Psychology, University of Wisconsin-Milwaukee, Milwaukee, WI, USA.; 84 Department of Pediatric Infectious Disease, Avera McKennan University Health Center, University of South Dakota, Sioux Falls, SD, USA.; 85 Department of Pediatrics, University of Toronto, Labatt Family Heart Center, The Hospital for Sick Children, Toronto, ON, Canada.; 86 Department of Pediatrics, University of Nebraska Medical Center, Omaha, NE, USA.; 87 Department of Pediatrics, Division of Developmental-Behavioral Pediatrics, New York University Grossman School of Medicine, New York, NY, USA.; 88 Department of Obstetrics and Gynecology, University of Utah Health, Salt Lake City, UT, USA.; 89 Department of Pediatrics, Valley Children’s Healthcare, Department of Pediatrics, Madera, CA, Madera, CA, USA.; 90 Department of Psychiatry, Virginia Commonwealth University, Richmond, VA, USA.; 91 Department of Pediatrics, New York Medical College, Valhalla, NY, USA.; 92 Department of Pediatrics, Section of Infectious Diseases and Global Health, Yale University School of Medicine, New Haven, CT, USA.; 93 Department of Pediatric Cardiology, Children’s Healthcare of Atlanta, Atlanta, GA, USA.; 94 Department of Pediatrics, Division of Pediatric Cardiology, Riley Hospital for Children, Indiana University School of Medicine, Indianapolis, IN, USA.; 95 Department of Pediatrics, University of New Mexico, Health Sciences Center, Albuquerque, NM, USA.; 96 Department of Pediatrics, Division of Infectious Diseases, Epidemiology and Hospital Medicine, University of Colorado Anschutz Medical Campus, Aurora, CO, USA.; 97 Department of Pediatrics, University of Michigan Health System, Ann Arbor, MI, USA.; 98 Department of Pediatric Cardiology, Phoenix Children’s Hospital, Phoenix, AZ, USA.; 99 Division of Pediatric Cardiology, Children’s Hospital of Michigan, Detroit, MI, USA.; 100 Department of Pediatric Infectious Disease, University of Nebraska Medical Center, Omaha, NE, USA.; 101 Department of Pediatrics, Children’s Hospital of Richmond at Virginia Commonwealth University, Richmond, VA, USA.; 102 Department of Pediatrics, Pediatric Cardiology, University of Mississippi Medical Center, Jackson, MS, USA.; 103 Department of Pediatrics, University of Texas Southwestern Medical Center, Dallas, TX, USA.; 104 Department of Pediatrics, University of Louisville School of Medicine, Louisville, KY, USA.; 105 Division of Cardiology, Children’s Hospital Los Angeles, Los Angeles, CA, USA.; 106 Department of Pediatrics, Avera McKennan Hospital and University Health Center, Sioux Falls, SD, USA.; 107 Nemours Cardiac Center, Nemours Childrens Health, Delaware, Wilmington, DE, USA.; 108 RECOVER Neurocognitive and Wellbeing/Mental Health Team, NYU Grossman School of Medicine, New York, NY, USA.; 109 Center for Individualized Medicine, Mayo Clinic Hospital, Rochester, MN, USA.; 110 Departments of Pediatrics and Population Health, New York University Grossman School of Medicine, New York, NY, USA.; 111 Department of Pediatrics, Connecticut Children’s Medical Center, Hartford, CT, USA.; 112 Department of Communication Sciences & Disorders, Northeastern University, Boston, MA, USA.

## Abstract

**Importance:**

The prevalence, pathophysiology, and long-term outcomes of COVID-19 (post-acute sequelae of SARS-CoV-2 [PASC] or “Long COVID”) in children and young adults remain unknown. Studies must address the urgent need to define PASC, its mechanisms, and potential treatment targets in children and young adults.

**Observations:**

We describe the protocol for the Pediatric Observational Cohort Study of the NIH’s **RE**searching **COV**ID to **E**nhance **R**ecovery (RECOVER) Initiative. RECOVER-Pediatrics is an observational meta-cohort study of caregiver-child pairs (birth through 17 years) and young adults (18 through 25 years), recruited from more than 100 sites across the US. This report focuses on two of five cohorts that comprise RECOVER-Pediatrics: 1) a *de novo* RECOVER prospective cohort of children and young adults with and without previous or current infection; and 2) an extant cohort derived from the Adolescent Brain Cognitive Development (ABCD) study (*n*=10,000). The *de novo* cohort incorporates three tiers of data collection: 1) remote baseline assessments (Tier 1, n=6000); 2) longitudinal follow-up for up to 4 years (Tier 2, n=6000); and 3) a subset of participants, primarily the most severely affected by PASC, who will undergo deep phenotyping to explore PASC pathophysiology (Tier 3, n=600). Youth enrolled in the ABCD study participate in Tier 1. The pediatric protocol was developed as a collaborative partnership of investigators, patients, researchers, clinicians, community partners, and federal partners, intentionally promoting inclusivity and diversity. The protocol is adaptive to facilitate responses to emerging science.

**Conclusions and Relevance:**

RECOVER-Pediatrics seeks to characterize the clinical course, underlying mechanisms, and long-term effects of PASC from birth through 25 years old. RECOVER-Pediatrics is designed to elucidate the epidemiology, four-year clinical course, and sociodemographic correlates of pediatric PASC. The data and biosamples will allow examination of mechanistic hypotheses and biomarkers, thus providing insights into potential therapeutic interventions.

## Introduction

The coronavirus disease 2019 (COVID-19) pandemic has significantly impacted child health. Nearly 100 million people have been diagnosed with COVID-19 in the United States (US), with nearly 16 million children [1]. Although it is estimated that between 10% and 30% of adults experience persistent symptoms from COVID-19 [2], termed post-acute sequelae of SARS-CoV-2 (PASC) or Long COVID, the prevalence in children is less well-established [3, 4]. As an emerging illness, the absence of universally-accepted PASC definitions in children challenge the elucidation of its epidemiology.

Unique challenges in understanding PASC symptoms in children have likely contributed to the limited evidence. Given that young children might not be able to articulate symptoms, studies must rely on caregiver interpretation. In addition, manifestation of symptoms may vary substantively across stages of physiological, emotional, and cognitive development [5]. As the medical community shifts from managing serious acute disease to addressing long-term consequences, large scale studies are needed to define and recognize PASC in children across the life course, to understand its natural history, and to develop evidence to guide successful treatment.

The pandemic began with a misconception that children were spared [6]. We now recognize that children and families are greatly impacted during both acute and chronic phases [7–10]. One distinct manifestation in children was recognized in April 2020; now called Multisystem Inflammatory Syndrome in Children (MIS-C) [11]. This debilitating hyperinflammatory syndrome has impacted over 9,000 children and young adults in the US [12], and represents a distinct post-acute syndrome that is typically recognizable in clinical practice. Other more chronic manifestations of PASC are challenging to characterize and identify. Furthermore, children with PASC may present with different symptoms and greater mental health concerns than adults [1, 13–19]. Additional phenotypes of childhood PASC are being reported, including phenotypes similar to postural orthostatic tachycardia syndrome (POTS), myalgic encephalomyelitis/chronic fatigue syndrome (ME/CFS), postintensive care unit syndrome, and potentially many others [20–22]. Therefore, a compelling rationale exists to invest resources and effort to study PASC in children. The NIH’s REsearching COVID to Enhance Recovery (RECOVER) Initiative responded by bringing together researchers, communities, and families in a systematic study of PASC in children [23]. Evidence that leads to improved health trajectories of children with PASC, could have population-level health impacts for decades to come.

### Study rationale

RECOVER has established the Pediatric Observational Cohort Study (RECOVER-Pediatrics), which is a combined retrospective and prospective longitudinal study, including five distinct cohorts, integrated together as a meta-cohort [23]. The overall goal is to characterize the clinical course, underlying mechanisms and long-term health effects of PASC on children and young adults from birth through 25 years old, to inform future pediatric preventive and treatment measures.

### Study aims

RECOVER-Pediatrics scientific aims are to:

1. Characterize the prevalence and incidence of new onset or worsening symptoms related to PASC
2. Characterize the spectrum of clinical symptoms of PASC, including distinct phenotypes, and describe the clinical course and recovery.
3. Identify risk and resiliency factors for developing PASC and recovering from PASC.
4. Define the pathophysiology of PASC, including subclinical organ dysfunction, and identify biological mechanisms underlying the pathogenesis of PASC.

## Materials and Methods

### Overview of study design

RECOVER-Pediatrics is a longitudinal, observational meta-cohort study of children and young adults (ages birth through 25 years) and their caregivers, recruited from healthcare- and community-based settings in more than 100 sites throughout the US, including Puerto Rico. Those *with and without* a history of a SAR-CoV-2 infection are included. For those 17 years or younger, data are collected by caregiver report and child direct assessments, and for those 18 through 25 years old by self-report. The study is being conducted from March 2022 to March 2026.

**Fig 1:**
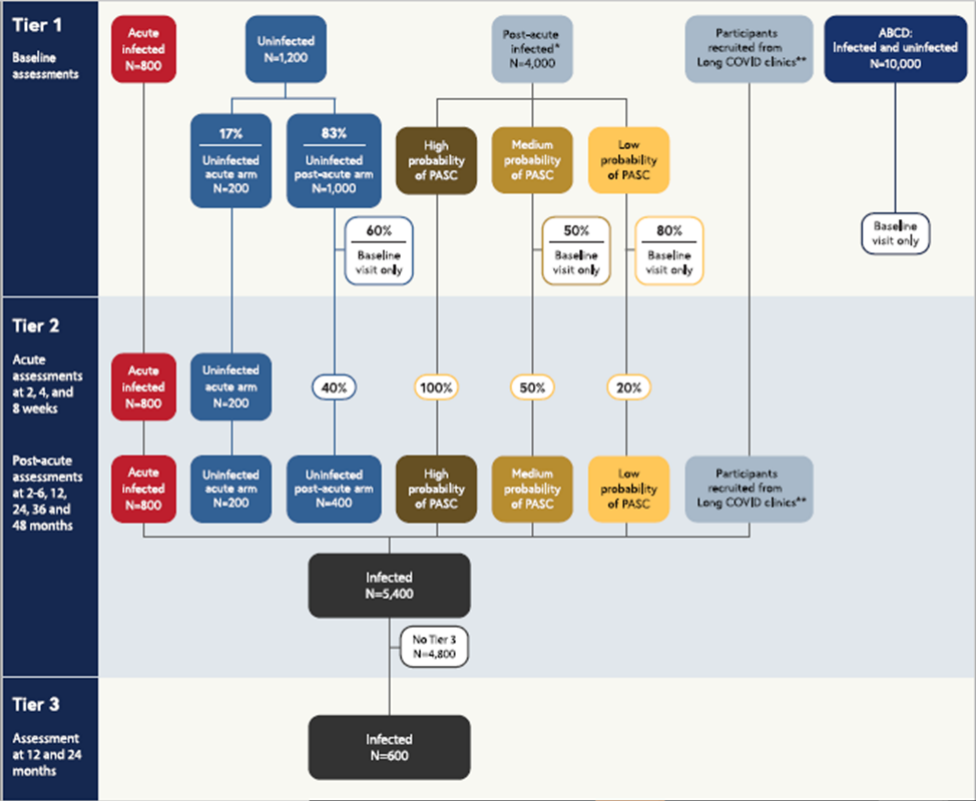
Overview of RECOVER-Pediatrics (de novo and ABCD cohorts).

The pediatric meta-cohort is comprised of five distinct cohorts: 1) *de novo RECOVER prospective cohort* including children and young adults ages birth through 25 years, with or without a known history of infection, and their caregivers; 2) *Adolescent Brain Cognitive Development (ABCD)* extant cohort, the largest long-term US study of brain development in adolescence [24, 25]; 3) *In utero exposure cohort*, including children less than 3 years old born to individuals with and without a SAR-CoV-2 infection during pregnancy [26, 27]; 4) *COVID MUSIC Study* extant cohort (*Long-Term Outcomes after the Multisystem Inflammatory Syndrome In Children*), including children and young adults with history of MIS-C [28]; and 5) cohort of children and young adults with a history of *post-COVID vaccine myocarditis*. This report focuses on the *de novo* cohort and ABCD (Figure 1).

Figure 1 shows a tiered overview of 2 of the 5 cohorts included in the meta-cohort (*de novo* RECOVER prospective cohort and ABCD), their participation in the three study tiers, and their targeted sample sizes (see Study Participants). Children and young adults ages newborn through 25 years old will be enrolled in the meta-cohort at Tier 1 for the *de novo* RECOVER prospective cohort (more than 6,000 from birth through 25 years old, including those with and without history of infection), and from ABCD (up to 10,000 adolescents with and without history of infection). All children and young adults enrolled in the study complete a baseline assessment (Tier 1). Percentages shown indicate random sampling proportions. Children and young adults without history of infection are assigned at random with prespecified proportions to the acute and post-acute arms. All children and young adults with history of infection who enroll into the acute arm and those without a history of infection who are randomized to the acute arm are asked to complete assessments at 2, 4, and 8 weeks. Following a promotion algorithm (Table 2), children and young adults in Tier 1 will be selected to be promoted to Tier 2, which includes assessments at 2–6, 12, 24, 36, and 48 months after enrollment. 600 children and young adults with history of infection, selected from Tier 2, will complete more intensive Tier 3 assessments at 12 and 24 months after enrollment.

*Children and young adults with history of infection who enroll in the post-acute arm (“post-acute infected”, n=4,000) are stratified into High, Medium, and Low probability of PASC groups based on a combination of past Long COVID diagnoses, Tier 1 Global PROMIS health measures, and symptom survey screener responses. Then, 100% of the high probability group, 50% of the medium probability group, and 20% of the low probability group are promoted at random to Tier 2. Since the distribution of these probability groups is unknown a priori, sample sizes are not specified for each category. Overall, the number of children and young adults who progress to Tier 2 will be less than the initial post-acute infected sample size, but the total target sample size for infected children and young adults in Tier 2 is 5,400.

** In order to achieve a sample of 5,400 children and young adults with history of infection in Tier 2 that is skewed towards those with greater likelihood of having PASC, additional children and young adults will be recruited from Long COVID clinics and subspecialty services to complete both Tier 1 and Tier 2 assessments.

RECOVER-Pediatrics is structured in a sequential fashion with three Tiers of data collection. Participants may be enrolled initially into Tier 1, which consists of a broad screening of health using remote surveys and biospecimen collection. Participants may subsequently progress to Tier 2, which includes a detailed review of health collected longitudinally for up to four years, using a combination of remote surveys and in-person assessments of biological and psychosocial data. In order to achieve the sample size required for Tier 2 assessments, other study participants will be recruited who present with a high probability of having PASC, such as those directly recruited from a clinic that focuses on Long COVID or presenting with a physician diagnosis of PASC. These participants will receive Tier 1 assessments and progress directly to Tier 2. Finally, in Tier 3, a subset of children and young adults most severely affected by PASC will undergo deep phenotyping with more intensive assessments to study PASC pathophysiology.

RECOVER-Pediatrics Tier 1 assessments aim to characterize the prevalence and incidence of new onset or worsening of sustained COVID-related symptoms (aim 1) and to gain a comprehensive understanding of the impact of exposure to a SARS-CoV-2 infection on broad physical, behavioral and mental health (aim 2). Tier 2 facilitates studying the natural history of PASC symptoms and potential recovery over time (aim 2). Child, household, and caregiver factors gathered in Tier 1, such as social determinants of health and prior health conditions, will be assessed to determine how they increase the risk of or protect against specific clinical outcomes (aim 3). Finally, Tier 3 data investigates long-term effects on multiple organ systems and child development (aim 4). Additionally, integration of Tier 1 and Tier 2 data will allow investigation of COVID-disease exposures and experiences which may be responsible for the clinical patterns observed in Tier 3.

The pediatric protocol was designed through collaboration across key stakeholders, including patients, caregivers, researchers, clinicians, community partners, and federal partners, fostering a patient-centered approach and promoting inclusivity and diversity. The pediatric protocol is adaptive to facilitate the changes needed in light of emerging science and the evolving pandemic.

### Study organizational structure and management

Study infrastructure includes four cores: 1) Clinical Science Core (CSC) at the NYU Grossman School of Medicine, which oversees study sites and provides scientific leadership in collaboration with hub and site Principal Investigators; 2) Data Resource Core (DRC) at Massachusetts General Hospital and Brigham and Women’s Hospital, which provides scientific and statistical leadership, and handles data management and storage; 3) PASC Biorepository Core (PBC) at Mayo Clinic, which manages biospecimens obtained; and 4) Administrative Coordinating Center (ACC) at RTI International, which provides operational and administrative support; collectively these form the Core Operations Group. The four cores are supported by oversight committees and pathobiology task forces provide content-specific input. RECOVER cohort studies are overseen by the National Community Engagement Group (NCEG) composed of patient and community representatives, a Steering Committee composed of site Principal Investigators and NIH program leadership, an Executive Committee composed of NIH Institute leadership, and an Observational Safety Monitoring Board composed of experts in longitudinal observational studies, epidemiology, bioethics, and biostatistics. RECOVER-Pediatrics includes 10 hubs that manage ~100 sites (Supplemental Table 1). NYU Grossman School of Medicine serves as the sIRB for sites without prior reliance agreements.

### Recruitment, consent, and screening strategies

The *de novo RECOVER prospective cohort study* is recruiting participants from healthcare- and community-based settings. Healthcare-based recruitment involves local media, text messaging, hospital websites, COVID registries, and partnerships with pediatric practices, nurse hotlines, or emergency departments. Community-based recruitment includes partnering with community health workers, school nurses, sports coaches, health fairs, and a mobile van to access rural communities. Participants can also join by self-referral through the RECOVER website, or in response to plain language and picture-based recruitment materials in both English and Spanish, which were developed with community input and using health literacy principles [29].

Eligible dyads complete an informed consent or assent process at enrollment for Tiers 1 and 2 (in-person or via electronic informed consent [e-consenting]). Young adults, aged 18 through 25 years old, sign their own informed consent. Tier 3 consent forms will only be completed when testing is offered.

In ABCD, 11,880 children aged 9–10 years old were recruited from community and school sites to participate in a 10-year study with the goal of understanding neurocognitive development during adolescence [24, 25, 30]. All ABCD participants are being contacted and offered enrollment into RECOVER-Pediatrics.

### Eligibility criteria

Children and young adults from birth through 25 years old are eligible to be enrolled in the *de novo cohort*, regardless of history of SARS CoV-2 infection. Enrolled participants are then categorized as either “infected” or “uninfected”: Infected participants have history of suspected, probable, or confirmed SARS-CoV-2 infection, defined by the World Health Organization (WHO) criteria [31], evidence of infection by serum antibody profile, or a history of MIS-C. Uninfected participants are those who self-report as having no history of a SARS-CoV-2 infection and who have never met WHO criteria; they have no evidence of a past asymptomatic infection in their medical history or evidence of past infection by serum antibody profile.

A primary caregiver, defined as an individual responsible for the enrolled child or young adult who resides in the same household, such as biological or nonbiological family member, is invited to enroll.

The primary exclusion criterion is any child or young adult with co-morbid illness with expected survival of less than 2 years. There is no limit to the number of children or young adults who can be enrolled from a single household. See supplemental tables for detailed eligibility criteria, definitions of analytic groups, and the World Health Organization Criteria (Supplement Tables 2, 3, and 4, respectively).

### Study participants

Recruitment is striving for a diverse sample that generally represents the US population, and encourages participation from rural or medically underserved communities, non-English speaking participants, and non-hospitalized participants with an acute COVID-19 infection. Participants are compensated for completing assessments and reimbursed for excess travel.

At least 6,000 participants will be recruited into the *de novo* cohort (Figure 1). Children and young adults with history of infection are classified into *one of two study arms* (acute arm vs. post-acute arm), based on their history of SARS-CoV-2 infection and infection dates. The *acute arm* includes 800 children and young adults whose most recent SARS-CoV-2 infection was 30 days or less prior to enrollment. The *post-acute arm* includes 4,000 children and young adults whose most recent SARS-CoV-2 infection was greater than 30 days prior to enrollment. In the group without a history of infection, 1,200 children and young adults will be randomly assigned to follow either the acute (200, or 17%) or post-acute (1,000 or 83%) arm of the protocol. Additional children and young adults will be recruited from Long COVID clinics and other subspecialty services in order to achieve Tier 2 sample size targets (see Timing of Study Assessments).

Up to 10,000 participants will also be recruited from the ABCD cohort.

### Timing of study assessments

The assessments for the *de novo cohort* consists of three tiers, which vary in timing, collection methods and intensity.

*Tier 1* (baseline visit for all participants) includes a single visit that is completed either via self-administration (remote and electronic) or research staff-assisted collection (e.g., telephone, videoconference, or in-person).

*Tier 2* (follow-up visits) includes five longitudinal in-person visits at 2 to 6-, 12-, 24-, 36- and 48-months post-enrollment. The children and young adults followed longitudinally in Tier 2 are selected based on a sampling scheme that prioritizes the acute arm as well as children and youth in the post-acute arm with a greater likelihood of having PASC. Promotion to Tier 2 occurs as follows: 1) All children/young adults in the *acute arm* with or without history of infection will be promoted; 2) children/young adults in the *post-acute arm with a history of infection* will be promoted at a rate dependent on their likelihood of PASC based on prior Long COVID diagnoses, Tier 1 PROMIS global health measure responses [32–34], and symptoms screener survey responses [16, 35] (Table 1); and 3) 40% of children/young adults without known infection in the post-acute arm, selected at random, will be promoted. In addition to promoting children and young adults from Tier 1, children and young adults will also be recruited from Long COVID clinics and subspecialty services to achieve the target sample size in Tier 2 of 6,000. These children and young adults will complete both Tier 1 and Tier 2 assessments. See Table 2 for a full description of the promotion algorithm.

Children and young adults in the acute arm with a history of infection will also complete remote assessments at 2, 4, and 8 weeks after their infection onset, with additional in-person assessments at 8 weeks. Children and young adults in the acute arm without history of infection will complete the same assessments, timed relative to their enrollment date. All ABCD youth are eligible to participate in RECOVER Tier 1, and can be referred to a *de novo* cohort site to participate in Tiers 2 and 3, if geographically feasible.

*Tier 3* has the most clinically intensive assessments with longitudinal in-person visits for a subset at 12 and 24 months post-enrollment. Tier 3 will include 600 children and young adults with history of infection from Tier 2.

### Main categories of data

Data collected for the *de novo* and ABCD cohorts are described below (Table 3).

*Surveys* include validated surveys with NIH common data elements, as available, informed by expert opinion (Supplemental Table 5). All are completed using Research Electronic Data Capture (REDCap), with the child’s first name coded within surveys to personalize the experience and to clarify which child the questions refer to given caregivers can have multiple children enrolled. For youth 17 years or younger, the caregiver is the primary respondent. Participants 18 through 25 years old are the primary respondent. Surveys assess sociodemographic information [36], child birth history [37], special health care needs [37–39], SARS-CoV-2 infection history, related conditions (e.g., MIS-C, POTS or other form of dysautonomia, and Long COVID diagnoses), COVID testing and vaccine history, COVID-related symptoms (both acute and long-term), COVID health consequences (e.g., diet [40], physical activity [40], sleep [40], screen time [40], schooling, parenting [41]) and social determinants of health (e.g., food insecurity [42], social support [43]). A list of potential Long COVID symptoms are assessed [16, 35] (Table 1), with respondents asked whether a specific problem or symptom is/was present for at least 4 weeks since the beginning of the COVID-19 pandemic and, for respondents with a history of infection, if the symptoms started before or after their infection.

*Clinical assessments* are completed at in-person Tier 2 visits across overarching domains of physical growth, physical health, neurocognition, and neurobehavioral function (Supplemental Table 6). Physical health domains include anthropometrics, vital signs, an active standing test measuring orthostatic blood pressures [44, 45], joint flexibility tests [46], electrocardiograms, and spirometry. Neurocognitive and neurobehavioral assessments vary by age (Table 4). Neurocognitive domains include broad and specific measures of attention, memory, receptive and expressive language skills, reading, and sensory function [47–51]. Neurobehavioral domains include a broad assessment of behavioral function including anxiety, mood, social interactions, aggression, sleep, self-regulatory behaviors, somatic complaints and attention concerns [52–59]. Tier 3 assessments follow the same domains, but provide more in-depth measurements. The promotion algorithm for Tier 3 is still under development. Physical health domains of cardio-pulmonary function are assessed by echocardiogram, cardiopulmonary exercise testing, cardiac MRI, pulmonary function tests, and sputum induction. Gastrointestinal function is assessed using abdominal ultrasound, and neurological function is assessed using brain MRI, electroencephalogram, and measures of neurocognitive function and psychiatric symptoms. These assessments include higher level measurement of all cognitive domains (thinking, language processing, memory, attention, and executive functioning) [60], visual motor integration and speed [61–63], and a psychiatric symptom battery [64].

*Biospecimens* are collected across all Tiers using kits designed specifically for each visit, timepoint, and participant age (Table 5; Supplemental Table 6). Tier 1 biospecimens consist of saliva and whole blood. Kits are shipped to homes for remote collection. Child and primary caregivers provide both saliva and blood; the other biological parent when available provides only saliva. Saliva is collected using Oragene devices (OGR-600) and banked for future DNA analysis. Whole blood is collected using a TASSO M20 device [65], which collects capillary blood using 4 volumetric sponges that each hold 17.5µL of blood (70 µL total). One sponge is used for SARS-CoV-2 spike and nucleocapsid antibody testing and remaining sponges are banked for future use.

Tier 2 acute biospecimens include saliva (Oragene OGR-600) and whole blood collections. All post-acute Tier 2 biospecimens consist of whole blood. The maximum amount of blood drawn at a single visit is age dependent. Whole blood is collected using serum separator tube (SST) and Ethylenediaminetetraacetic tube acid (EDTA) across all ages above 24 months and an additional cell preparation tube (CPT) is included at participants 6 years of age and older.

Tier 3 biospecimens consist of whole blood, sputum, swabs (e.g., skin, nasal, oral), urine and stool. Collection of Tier 3 biospecimens is limited to children ages 3 years and older (maximum allowable volume is age dependent).

### Statistical methods

We will estimate the proportion of children and young adults experiencing new onset or worsening of each symptom (incidence), stratified by age (0–2, 3–5, 6–12, 13–17, 18–25 years), over time. Prevalence within the recruited population will be estimated by calculating the point prevalence of each symptom at each 3-month interval since infection by calculating the proportion of children and young adults who are currently experiencing each symptom at each study visit. The excess burden of each symptom due to infection will be assessed by calculating differences in incidence and prevalence between children and young adults with and without an infection history. Odds ratios and relative risks for the association between infection and onset of each symptom will also be calculated, adjusting for sex in each age strata.

A preliminary case definition of PASC will be informed by using variable selection methods to identify which symptoms best differentiate children and young adults with and without an infection. The estimated associations obtained from regression models will be used to define a PASC score, with a cutoff for PASC defined based on clinical expertise while ensuring that the rate of those with no history of infection who are diagnosed as having PASC is reasonably low. This preliminary symptom-based case definition will be modified and augmented by clinical and subclinical findings as they become available. To identify PASC phenotypes among children and young adults who are classified as having PASC defined by symptom patterns, we will use unsupervised learning methods to discover symptom clusters within each age strata (e.g., agglomerative hierarchical clustering [66] and consensus clustering [67]) to define PASC sub-phenotypes.

With this definition of PASC, we will conduct regression analyses to evaluate whether the risk of PASC and PASC sub-types differs by multiple factors, including demographic, clinical, and caregiver characteristics, social determinants of health, SARS-CoV-2 infection and immunization history, symptom severity during the acute phase of SARS-CoV-2 infection, and therapeutic exposures. Among participants in Tier 2 who develop PASC, we will use time-to-event analyses to identify factors that influence time to recovery from PASC. To investigate biomarkers related to PASC, clinical laboratory assessments will be compared between children and young adults who do and do not develop PASC. Mediation analyses will also be used to study the pathways by which SARS-CoV-2 infection leads to the development of PASC.

### Power calculations

Power calculations for the *de novo cohort* were performed prior to recruitment using a type 1 error rate of 0.01. With 4,800 infected and 1,200 uninfected children and young adults from both acute and post-acute arms in Tier 1, assuming the risk of a given symptom in the uninfected group is 10%, we have 90% power to detect a difference as small as 4.1% in the frequency of that symptom between groups.

In Tier 2, given the sampling and promotion framework described in *Timing of Study Assessments*, our sample with longitudinal follow-up will be skewed towards those who are more likely to have PASC. Following development of a definition of PASC, we consider the scenario in which we assume that of the 5,400 children and young adults with a history of infection in Tier 2, 3,600 meet PASC criteria and 1,800 do not. For a hypothetical risk factor with 50% prevalence in the PASC- group, we have 90% power to detect an odds ratio as small as 1.25 for the odds of PASC for those with the risk factor versus those without. For a factor with 25% prevalence in the PASC-negative group, the minimum detectable odds ratio is 1.29. In our Tier 3 sample of 600 children and young adults with history of infection (which includes additional data on biomarkers), assuming the sample has 400 with PASC and 200 without PASC, for a marker with 10% prevalence in the PASC- group, we have 90% power to detect an odds ratio as small as 2.60 for PASC.

## Discussion

The overall goal of RECOVER-Pediatrics is to improve our understanding of recovery after SARS-CoV-2 infection, with a focus on the prevalence, natural history, and pathogenesis of PASC in children and young adults. Successful completion should lead to formal characterization of pediatric PASC as its own syndrome. This is essential to develop diagnostic, treatment, and preventive strategies tailored to children’s unique physiology.

RECOVER-Pediatrics is well positioned to ascertain the epidemiology, four-year clinical course, and sociodemographic contributions to pediatric PASC, with rich data and biosamples available to readily test further mechanistic hypotheses, establish biomarkers, and provide insights into potential therapies. The meta-cohort is designed to provide details that are not available in other large epidemiologic or electronic health records queries, including a dynamic study design that can be flexible and responsive as new variants arise, and as our understanding of the long-term effects of SARS-CoV-2 evolves. RECOVER-Pediatrics was designed to include a wide range of ages, and diverse socioeconomic, racial, ethnic and geographic populations to ensure that findings are generalizable, and provide equitable benefit for all.

The generation-defining nature of the COVID-19 pandemic will impact the life course of children in ways that we have yet to fully understand. The unprecedented scope of RECOVER-Pediatrics sets the stage for not only characterizing a new disorder that will impact children for years to come, but also for identifying and deploying solutions through its collaborations with investigators and communities across the country.

RECOVER-Pediatrics is expected to gather a rich data set that can be used to develop treatments for persons with Long COVID and provide guidelines for how to respond more quickly to prevent, reduce the consequences, and treat complications of future coronavirus outbreaks which are likely to emerge.

## Supplementary Material

Supplement 1

## Figures and Tables

**Table 1:** Potential Post-Acute Sequelae of SARS-CoV-2 (PASC) Symptoms Being Assessed in RECOVER-Pediatrics*

Major or Minor Classification**	Symptoms being Assessed
**General symptoms or problems**	
**Major**	Fever
	Feeling sleepy during the day^a^
	Fussy or cranky (crying a lot)^b^
	Low energy or not feeling strong enough to do things^a^
	Feeling very tired all day long^a^
	Feeling very tired after walking^a^
	Not wanting to eat (poor appetite)
	Lost weight or gained less than expected
	Lost height or grew less than expected
**Minor**	Trouble sleeping
	Hot and cold spells (feeling hot or cold for no reason)^a^
	Sweating more than normal
	Wanting to eat more than normal (increased appetite)
	Wanting to drink liquids more than normal (increased thirst)
	Gained weight more than expected
**Symptoms or problems in the eyes, ears, nose, and throat**	
**Major**	Light hurts your eyes^a^
	Change in hearing^a^
	Ringing in the ears^a^
	Change in smell^a^
	Loss of smell^a^
	Throat hurts (sore throat)^a^
	Loss of voice (sounding hoarse)
	Problems swallowing
	Change in how things taste^c^
**Minor**	Eyes look red
	Eyes are watery
	Eyes are dry
	Dark circles or color under the eyes
	Trouble seeing or blurry vision^a^
	Stuffy nose or runny nose
	Very dry mouth^a^
	Problems with teeth or gums
	Chapped lips
**Symptoms or problems involving the heart and lungs**	
**Major**	Dry cough
	Wet cough (brings up mucus)
	Barking cough
	Trouble breathing (breathing too fast)
	Pain when breathing^a^
	Pain in the chest^c^
	Feeling like your heart is beating really fast, racing, or pounding (called palpitations) when not doing exercise^c^
	Feeling like your heart is beating really fast when doing exercise^c^
	Fainting or feeling like you are going to faint (lightheaded) ^c^
	Trouble walking^a^
	Trouble climbing stairs^a^
	Trouble running^a^
**Symptoms or problems involving the belly**	
**Major**	Nausea (feeling like you are going to throw up)
	Throwing up (vomiting)
	Loose stool (diarrhea)
	Pain with peeing (urination)^a^
	Peeing more than normal (urination more than normal) ^a^
**Minor**	Stomach pains/cramps^a^
	Trouble pooping/stooling (constipation)
**Symptoms or problems involving the skin, hair, and nails**	
**Major**	Skin rash
	Changes or problems with nails
	Changes or problems with hair
	Color changes in your skin, such as red, white or purple
	Color changes on the fingers or toes
**Minor**	Itchiness of the skin^a^
**Symptoms or problems involving the bones and muscles**	
**Major**	Muscle weakness
	Pains in the joints (like the elbows, knees, ankles) ^a^
	Pain in the back^a^
	Pain in the neck^a^
**Minor**	Sore muscles or pain in the muscles^a^
	Body aches or pains
**Symptoms or problems involving the brain and nerves**	
**Major**	Headache^c^
	Feeling dizzy (feeling like the room is spinning)^c^
	Shakiness or tremors^c^
	Feeling tingling or ‘pin-and-needles’ in the hands and feet^c^
	Unable to move part of the body
	Problems with remembering things (memory)^a^
	Problems with focusing on things (concentration), sometimes called “brain fog” ^a^
	Problems with talking ^a^
**Symptoms or problems involving feelings or behavior**	
**Major**	Feeling sad or depressed^c^
	Feeling anxious or on edge^c^
	Feeling a lot of fear when being away from parent or caregiver^b^
	Feeling a lot of fear of specific things like spiders or being up high^d^
	Feeling a lot of fear about being with other children or adults^d^
	Feeling fear of crowds or being in closed-in spaces^c^
	Having a sudden intense feeling of fear, like a panic attack^e^
	Refusing to go to school^c^
	Seeing, hearing, or feeling that something is there when it is not (hallucinations)^c^
	Being hyperactive or much more active than other children^a^
	Refusing to follow rules or doing what they are asked to do^a^
	Serious breaking of rules like lying, stealing, starting fights, or bullying^a^
	Having repeating memories, dreams, thoughts, or worries after a traumatic event^a^
**Minor**	Having a lot of tantrums^b^
	Holding their breath for a long time when they are afraid or angry^b^
	Having nightmares
	Screaming in fear while asleep, sometimes called night terrors^b^
	Aggressive behavior like hitting, biting or kicking^b^
	Rocking the body back and forth or head banging
**Symptoms or problems involving periods**	
**Minor**	Getting periods less often ^f^
	Getting periods more often ^f^
	Heavier periods ^f^
	Lighter periods ^f^

* The following question is used to assess potential PASC symptoms in RECOVER-Pediatrics: “Did your child have any of these problems or symptoms lasting for more than 4 weeks that started or got worse since the COVID pandemic began in March 2020? These are problems or symptoms that kept happening without stopping or kept happening again and again for longer than 4 weeks.”

** Symptoms were classified as major or minor depending on their severity and presumed likelihood of being associated with a COVID-19 infection.

a Symptoms included for children 3 years or older.

b Symptom included for children 5 years or younger.

c Symptom included for children 6 years or older.

d Symptoms included for children 2 years or older.

e Symptoms included for children 12 years or older.

f Symptoms included for those who report having menses.

**Table 2.** Promotion Algorithm Used in the *de novo* RECOVER-Pediatrics Cohort for Selecting Children and Young Adults for the Longitudinal Follow-Up (Tier 2).

Type of participant	Subgroup	Subgroup criteria	Promotion rate to Tier 2
“*Acute infected”*: Children and young adults who reported having a COVID infection ≤30 days prior to enrollment	All	None	100%
“Post-a*cute infected”*: Children and young adults who reported having a COVID infection >30 days prior to enrollment	High probability of PASC^a^	Any of the following: 1. Prior diagnosis of Long COVID or MIS-C based on study site medical record review or referral by a health care provider 2. Recruitment from a Long COVID clinic 3. ≥1 fair/poor responses on the global health PROMIS scale^b^ and ≥1 major or minor symptom reported^c^ 4. ≥1 good/fair/poor responses on the global health PROMIS scale^b^ and ≥ 1 major symptom reported^c^ 5. ≥1 good/fair/poor responses on the global health PROMIS scale^b^ and ≥2 minor symptoms reported^c^	100%
	Medium probability of PASC^a^	Any of the following: 1. ≥1 good responses on the global health PROMIS scale^b^ and ≥1 major or minor symptom reported^c^ 2. ≥1 very good/good/fair/poor responses on the global health PROMIS scale^b^ and ≥1 major symptom reported^c^ 3. ≥1 very good/good/fair/poor responses on the global health PROMIS scale^b^ and ≥2 minor symptoms reported^c^	50%
	Low probability of PASC^a^	Does not meet high or medium probability of PASC criteria	20%
“*Uninfected”*: Children or Young adults	Acute arm	At enrollment, 17% of the “uninfected” group were randomly assigned to participate in the acute arm of the study.	100%
without a known history of a COVID infection	Post-acute arm	At enrollment, 83% of the “uninfected” group were randomly assigned to participare in the post-acute arm of the study. 40% of this group will be randomly assigned to participate in Tier 2	40%

a Responses to the PROMIS Global Health Scales and the presence of major and minor symptoms are used to categorize participants who are post-acute infected as high, medium, or low probability of PASC.

b The PROMIS Global Health Scales are self-reported or caregiver-reported measures of overall, physical, and mental health for young adults and children, respectively [32–34]. The three questions from the caregiver-reported version that are used in the algorithm, include: 1) “*In general, would you say your child’s health is?;* 2) “*In general, how would you rate your child’s physical health?*”; and 3) “*In general, how would you rate your child’s mental health, including mood and ability to think?*” Responses include: *Excellent, Very Good, Good, Fair, or Poor*.

c A list of all major and minor symptoms, reported at the enrollment visit as part of the symptom screener, is provided in the Table 1 [16, 35]. Not all symptoms are asked of all participants, as many are age-specific (e.g., fewer symptoms assessed for younger children) and sex-specific (e.g., menses related symptoms).

**Table 3:** Summary of Study Assessments in the *de novo* RECOVER-Pediatrics Cohort^a^

	BL	…	2	4	8	…	2 to 6		12		24		36		48
			(weeks)				(months)								
Tier 1 and Tier 2 Assessments	BL		Acute phase				Post-acute phase								
Identity															
Demographics															
Child Birth History															
Child Current Health Status															
Special Health Care Needs Screener															
PROMIS Global Health															
First COVID Infection History															
Current COVID Infection History															
Weekly COVID Infection History															
COVID Infection History (Follow-up)															
Related Conditions (MIS-C, POTS)															
COVID Testing History															
COVID Family Infection															
COVID Symptoms															
COMPASS-31															
COVID Vaccine History															
COVID Health Consequences															
Social Determinants of Health															
Child Wellbeing															
Tier 1 Biospecimen															
Anthropometry & Vital Signs															
Electrocardiogram															
Spirometry															
Pulse Oximetry															
Tier 2 Acute Biospecimens															
10 Minute Active Standing Test (Orthostatic BP)															
Joint Flexibility (Beighton Scale)															
Neurocognitive Development (e.g., NIH Toolbox)															
Emotional/Mental Health															
Post-Acute Tier 2 Biospecimens															
**Tier 3 Assessments**															
Echocardiogram															
Cardiac MRI															
Pulmonary Function Tests (PFTs)															
Lung Microbiome (Sputum Induction)															
Cardiopulmonary Exercise Testing															
Abdominal Ultrasound															
Brain MRI															
Brain EEG															
Neurocognitive Testing															
Tier 3 Biospecimens															

a Blue=Questionnaires; Red=Clinical Assessments; Yellow=Biospecimen Collection

**Table 4:** Neurocognitive, Neurobehavioral, Well-Being and Mental Health Measures by Age in Tiers 2 and 3 for the *de novo* RECOVER-Pediatrics Cohort

Study Tier	Neurocognitive and Developmental Assessments	Neurobehavioral, Well-Being and Mental Health Assessments
**Infancy and Toddlerhood: Birth through 2 years old**		
Tier 2	Ages and Stages Questionnaire—3rd Edition (ASQ-3) (47–49) Modified Checklist for Autism in Toddlers Revised with Follow up (MCHAT-RF) (50)	Ages and Stages Questionnaires: Social-Emotional, 2nd Edition (ASQ:SE-2) (52) Child Behavior Checklist (53)
Tier 3	N/A	N/A
**Preschool-Age: 3 years old through 5 years old**		
Tier 2	Ages and Stages Questionnaire—3rd Edition (ASQ-3) (47–49) NIH Toolbox Cognitive Measures (51)	Ages and Stages Questionnaires: Social-Emotional, 2nd Edition (ASQ:SE-2) (52) Child Behavior Checklist (53) Patient-Reported Outcomes Measurement Information System (PROMIS^®^) Parent Proxy Anger Scale (54) PROMIS^®^ Parent Proxy Psychological Stress Experiences Scale (55) PROMIS^®^ Parent Proxy Positive Affect Scale (56)
Tier 3	*Cognitive*: Woodcock Johnson Cognitive Battery subtests (60) *Language*: Woodcock-Johnson Oral Language Battery subtests (60) *Verbal Memory*: Woodcock Johnson subtests (60) *Visual Memory*: Wide Range Assessment of Memory and Learning (61) *Visual-Motor Drawing*: Beery Buktenica (62) *Visual Motor Speed*: Purdue Pegboard (63) *Pre-Academics*: Woodcock-Johnson Achievement Battery subtests (60)	Kiddie SADS computer completed by caregiver (64)
**School-age and Adolescence: 6 years old through 17 years old**		
Tier 2	NIH Toolbox Cognitive Measures (51)	Patient-Reported Outcomes Measurement Information System (PROMIS^®^) Parent Proxy Anger Scale (54) PROMIS^®^ Parent Proxy Psychological Stress Experiences Scale (55) PROMIS^®^ Parent Proxy Positive Affect Scale (56) Revised Children’s Anxiety and Depression Scale (RCADS-25) (57) Strengths and Difficulties Questionnaire (Hyperactivity/Inattention and Conduct Problems Subscales) (58)
Tier 3	*Cognitive*: Woodcock Johnson Cognitive Battery subtests (60) *Language*: Woodcock-Johnson Oral Language Battery subtests (60) *Verbal Memory*: Woodcock Johnson subtests (60) *Visual Memory*: Wide Range Assessment of Memory and Learning (61) *Visual-Motor Drawing*: Beery Buktenica (62) *Visual Motor Speed*: Purdue Pegboard (63) *Pre-Academics*: Woodcock-Johnson Achievement Battery subtests (60)	Kiddie SADS computer completed by caregiver (64)
**Young Adults: 18 years through 25 years old**		
Tier 2	NIH Toolbox Cognitive Measures (51)	Patient-Reported Outcomes Measurement Information System (PROMIS^®^) Parent Proxy Anger Scale (54) PROMIS^®^ Parent Proxy Psychological Stress Experiences Scale (55) Achenbach Adult Self Report (59)
Tier 3	*Cognitive*: Woodcock Johnson Cognitive Battery subtests (60) *Language*: Woodcock-Johnson Oral Language Battery subtests (60) *Verbal Memory*: Woodcock Johnson subtests (60) *Visual Memory*: Wide Range Assessment of Memory and Learning (61) *Visual-Motor Drawing*: Beery Buktenica (62) *Visual Motor Speed*: Purdue Pegboard (63)	SADS Structured Psychiatric Interview (64)

**Table 5:** Biospecimen Collection and Processing Summary

Participant Samples Collected For	Collected Specimen	Quantity^a^	Biobanked Specimen Type	Number of Aliquots	Aliquot Volume
**Tier 1**					
Pediatric Participant; Primary Caregiver; Additional Biologic Parent	OGR-600 – Saliva	1 × 2mL	Saliva	NA	2 mL
Pediatric Participant; Primary Caregiver	TASSO M20 – Capillary Blood	1 × 70 µL	Blood	4 x volumetric sponges	17.5 µL
**Tier 2 Acute**					
All Age Groups over 24 months	Oragene 600 – Saliva	1 × 2mL	Saliva	N/A	2 mL
All Age Groups over 24 months	Serum Separator Tube (SST) – Whole Blood^b^	1 × 5mL	Serum	5	500 µL
All Age Groups over 24 months	EDTA – Whole Blood^c^	1 × 10mL	• Plasma • White Blood Cells (WBCs) • Red Blood Cells (RBCs)	• 13 x Plasma • 1 x WBC • 3 x RBCs	• 5 × 200 µL Plasma (Rutgers) • 8 × 500 µL Plasma (PBC) • WBC – 1 mL • RBCs – 1 mL
Ages 6–25 yrs.	Sodium Citrate Cell Preservation Tube (CPT) – Whole Blood^d^	2 × 4 mL	• Peripheral Blood Mononuclear Cells (PBMCs)	~3 x PBMCs (target cell count minimum 5 million cells/mL)	1 mL
**Tier 2 Post Acute**					
Ages 24mo - under 6 years (all post-acute visits)	Serum Separator Tube (SST) – Whole Blood^b^	1 × 5mL	Serum	5	• 500 µL
Ages 6–25 yrs (6 month visit only)	Serum Separator Tube (SST) – Whole Blood^b^	2 × 5mL	Serum	3	• 1 × 1 mL - ARUP • 1 × 2 mL - ARUP • 1 × 1.5 mL – PBC • 6 × 200 µL for Rutgers and/or • 3 × 500 µL for PBC
Ages 6–9 yrs.	Sodium Citrate Cell Preservation Tube (CPT) – Whole Blood^d^	2 × 4 mL	Peripheral Blood Mononuclear Cells (PBMCs)	8 x PBMCs (target cell count minimum 5 million cells/mL)	1 mL
Ages 10–25 yrs.	Sodium Citrate Cell Preservation Tube (CPT) – Whole Blood^d^	4 × 4 mL	Peripheral Blood Mononuclear Cells (PBMCs)	16 x PBMCs (target cell count minimum 5 million cells/mL)	1 mL
All Age Groups EXCEPT 6-month Post Acute visit for age 6–9 yrs	EDTA – Whole Blood^c^	1 × 10mL	• Plasma • White Blood Cells (WBCs) • Red Blood Cells (RBCs)	• 13 x Plasma • 1 x WBC • 3 x RBCs	• 5 × 200µL Plasma (Rutgers) • 8 × 500µL Plasma (PBC) • WBC – 1mL • RBCs – 1 mL
**Tier 3** ^e^					
All Age Groups	Serum Separator Tube (SST) – Whole Blood^b^	TBD	• Serum	• TBD	• TBD
All Age Groups	EDTA – Whole Blood^c^	TBD	• Plasma • White Blood Cells (WBCs) • Red Blood Cells (RBCs)	• TBD	• TBD
All Age Groups	Lithium Heparin – Whole Blood	TBD	• Plasma	• TBD	• TBD
All Age Groups	Red Top (No Additive) – Whole Blood	TBD	• Serum	• TBD	• TBD
All Age Groups	Other Biospecimens for Microbiome Analysis (e.g. sputum, swaps (skin, nasal, oral), urine, stool)	TBD	• Sputum • Swabs • Urine • Stool	• TBD	• TBD

a Sample volumes are age dependent: (newborn to under 6 years: maximum draw of 2 mL per kg of body weight; 6 to under 10 years: 25 mL; greater than 10 years old: 38 mL)

b SST tube is collected and within 4 hours of collection the SST tube is centrifuged, serum is aliquoted and frozen locally at collection sites. Serum aliquots are batch shipped frozen on dry ice in monthly intervals and are banked for future research.

c The EDTA tube is collected for all age groups and is processed for plasma, WBC, and RBC aliquots. A plasma aliquot is sent out for central testing. The other EDTA aliquot derivatives are frozen and banked for future research.

d The CPT tubes are only collected for age groups 6–25 years. The CPT tubes are centrifuged at collection sites and sent on ice packs day of collection to the PBC. Once arrived at the PBC, the CPT tubes are processed. A maximum of 8 × 1 mL PBMC aliquots (minimum of 5 million cells/mL) are derived. PBMC aliquots are stored in liquid nitrogen and banked for future research.

e Tier 3 biospecimen parameters are currently under development, but will involve collection of whole blood for clinical chemistry and biobanking and the collection and banking of biospecimens for microbiome analysis.

## References

[1] Davis HE, McCorkell L, Vogel JM, Topol EJ. Long COVID: major findings, mechanisms and recommendations. Nat Rev Microbiol. 2023;21(3):133–46. Epub 20230113. doi: 10.1038/s41579-022-00846-2. 36639608 PMC9839201

[2] World Health Organization. WHO Coronavirus (COVID-19) dashboard 2023 2023. Available from: https://covid19.who.int/.

[3] Zimmermann P, Pittet LF, Curtis N. How common is Long COVID in children and adolescents? Pediatr Infect Dis J. 2021;40(12):e482–e7. doi: 10.1097/inf.0000000000003328. 34870392 PMC8575095

[4] Kompaniyets L, Bull-Otterson L, Boehmer TK, Baca S, Alvarez P, Hong K, Post-COVID-19 symptoms and conditions among children and adolescents - United States, March 1, 2020-January 31, 2022. MMWR Morb Mortal Wkly Rep. 2022;71(31):993–9. Epub 20220805. doi: 10.15585/mmwr.mm7131a3. 35925799 PMC9368731

[5] Borch L, Holm M, Knudsen M, Ellermann-Eriksen S, Hagstroem S. Long COVID symptoms and duration in SARS-CoV-2 positive children - a nationwide cohort study. Eur J Pediatr. 2022;181(4):1597–607. Epub 20220109. doi: 10.1007/s00431-021-04345-z. 35000003 PMC8742700

[6] Forrest CB, Burrows EK, Mejias A, Razzaghi H, Christakis D, Jhaveri R, Severity of acute COVID-19 in children <18 years old March 2020 to December 2021. Pediatrics. 2022;149(4). doi: 10.1542/peds.2021-055765. 35322270

[7] Lopez-Leon S, Wegman-Ostrosky T, Ayuzo Del Valle NC, Perelman C, Sepulveda R, Rebolledo PA, Long-COVID in children and adolescents: a systematic review and meta-analyses. Sci Rep. 2022;12(1):9950. Epub 20220623. doi: 10.1038/s41598-022-13495-5. 35739136 PMC9226045

[8] Chua PEY, Shah SU, Gui H, Koh J, Somani J, Pang J. Epidemiological and clinical characteristics of non-severe and severe pediatric and adult COVID-19 patients across different geographical regions in the early phase of pandemic: a systematic review and meta-analysis of observational studies. J Investig Med. 2021;69(7):1287–96. Epub 20210616. doi: 10.1136/jim-2021-001858. PMC8485127 34135068

[9] Funk AL, Kuppermann N, Florin TA, Tancredi DJ, Xie J, Kim K, Post-COVID-19 conditions among children 90 days after SARS-CoV-2 infection. JAMA Netw Open. 2022;5(7):e2223253. Epub 20220701. doi: 10.1001/jamanetworkopen.2022.23253. 35867061 PMC9308058

[10] Groff D, Sun A, Ssentongo AE, Ba DM, Parsons N, Poudel GR, Short-term and long-term rates of Postacute Sequelae of SARS-CoV-2 Infection: A systematic review. JAMA Netw Open. 2021;4(10):e2128568. Epub 20211001. doi: 10.1001/jamanetworkopen.2021.28568. 34643720 PMC8515212

[11] Dufort EM, Koumans EH, Chow EJ, Rosenthal EM, Muse A, Rowlands J, Multisystem Inflammatory Syndrome in Children in New York State. N Engl J Med. 2020;383(4):347–58. Epub 20200629. doi: 10.1056/NEJMoa2021756. 32598830 PMC7346766

[12] Centers for Disease Control and Prevention (CDC). COVID data tracker 2023 [cited 2023 March 9]. Available from: https://covid.cdc.gov/covid-data-tracker/#mis-national-surveillance.

[13] Stephenson T, Pinto Pereira SM, Shafran R, de Stavola BL, Rojas N, McOwat K, Physical and mental health 3 months after SARS-CoV-2 infection (long COVID) among adolescents in England (CLoCk): a national matched cohort study. Lancet Child Adolesc Health. 2022;6(4):230–9. Epub 20220208. doi: 10.1016/s2352-4642(22)00022-0. 35143770 PMC8820961

[14] Hernandez-Romieu AC, Carton TW, Saydah S, Azziz-Baumgartner E, Boehmer TK, Garret NY, Prevalence of select new symptoms and conditions among persons aged younger than 20 years and 20 years or older at 31 to 150 days after testing positive or negative for SARS-CoV-2. JAMA Netw Open. 2022;5(2):e2147053. Epub 20220201. doi: 10.1001/jamanetworkopen.2021.47053. 35119459 PMC8817203

[15] Buonsenso D, Pujol FE, Munblit D, Pata D, McFarland S, Simpson FK. Clinical characteristics, activity levels and mental health problems in children with long coronavirus disease: a survey of 510 children. Future Microbiol. 2022;17(8):577–88. Epub 20220401. doi: 10.2217/fmb-2021-0285. 35360923 PMC9248023

[16] Kikkenborg Berg S, Dam Nielsen S, Nygaard U, Bundgaard H, Palm P, Rotvig C, Long COVID symptoms in SARS-CoV-2-positive adolescents and matched controls (LongCOVIDKidsDK): a national, cross-sectional study. Lancet Child Adolesc Health. 2022;6(4):240–8. Epub 20220208. doi: 10.1016/s2352-4642(22)00004-9. 35143771 PMC8820960

[17] Wulf Hanson S, Abbafati C, Aerts JG, Al-Aly Z, Ashbaugh C, Ballouz T, Estimated global proportions of individuals with persistent fatigue, cognitive, and respiratory symptom clusters following symptomatic COVID-19 in 2020 and 2021. Jama. 2022;328(16):1604–15. doi: 10.1001/jama.2022.18931. 36215063 PMC9552043

[18] Haddad A, Janda A, Renk H, Stich M, Frieh P, Kaier K, Long COVID symptoms in exposed and infected children, adolescents and their parents one year after SARS-CoV-2 infection: A prospective observational cohort study. EBioMedicine. 2022;84:104245. Epub 20220922. doi: 10.1016/j.ebiom.2022.104245. 36155957 PMC9495281

[19] Roessler M, Tesch F, Batram M, Jacob J, Loser F, Weidinger O, Post-COVID-19-associated morbidity in children, adolescents, and adults: A matched cohort study including more than 157,000 individuals with COVID-19 in Germany. PLoS Med. 2022;19(11):e1004122. Epub 20221110. doi: 10.1371/journal.pmed.1004122. 36355754 PMC9648706

[20] Brugler Yonts A. Pediatric Long-COVID: A review of the definition, epidemiology, presentation, and pathophysiology. Pediatr Ann. 2022;51(11):e416–e20. Epub 20221101. doi: 10.3928/19382359-20220913-06. 36343180

[21] Rao S, Lee GM, Razzaghi H, Lorman V, Mejias A, Pajor NM, Clinical features and burden of Postacute Sequelae of SARS-CoV-2 Infection in children and adolescents. JAMA Pediatr. 2022;176(10):1000–9. doi: 10.1001/jamapediatrics.2022.2800. 35994282 PMC9396470

[22] Morrow AK, Malone LA, Kokorelis C, Petracek LS, Eastin EF, Lobner KL, Long-term COVID 19 sequelae in adolescents: the overlap with orthostatic intolerance and ME/CFS. Curr Pediatr Rep. 2022;10(2):31–44. Epub 20220309. doi: 10.1007/s40124-022-00261-4. 35287333 PMC8906524

[23] National Institutes of Health. RECOVER: Researching COVID to Enhance Recovery 2023 [cited 2023 March 12]. Available from: https://recovercovid.org/.

[24] Barch DM, Albaugh MD, Avenevoli S, Chang L, Clark DB, Glantz MD, Demographic, physical and mental health assessments in the adolescent brain and cognitive development study: Rationale and description. Dev Cogn Neurosci. 2018;32:55–66. Epub 20171103. doi: 10.1016/j.dcn.2017.10.010. 29113758 PMC5934320

[25] Volkow ND, Koob GF, Croyle RT, Bianchi DW, Gordon JA, Koroshetz WJ, The conception of the ABCD study: From substance use to a broad NIH collaboration. Dev Cogn Neurosci. 2018;32:4–7. Epub 20171010. doi: 10.1016/j.dcn.2017.10.002. 29051027 PMC5893417

[26] Eunice Kennedy Shriver National Institute of Child Health and Human Development. Maternal-Fetal Medicine Units Network 2023. Available from: https://www.nichd.nih.gov/research/supported/mfmu. 10.1016/j.ajog.2008.06.021 18674652

[27] The Regents of the University of California. PRIORITY: Pregnancy Coronavirus Outcomes Registry University of California, San Francisco 2023. Available from: https://priority.ucsf.edu/.

[28] Truong DT, Trachtenberg FL, Pearson GD, Dionne A, Elias MD, Friedman K, The NHLBI Study on Long-terM OUtcomes after the Multisystem Inflammatory Syndrome In Children (MUSIC): Design and objectives. Am Heart J. 2022;243:43–53. Epub 20210819. doi: 10.1016/j.ahj.2021.08.003. 34418362 PMC8710361

[29] Davis TC, Gazmararian J, Kennen EM. Approaches to improving health literacy: lessons from the field. J Health Commun. 2006;11(6):551–4. doi: 10.1080/10810730600835517. 16950727

[30] Garavan H, Bartsch H, Conway K, Decastro A, Goldstein RZ, Heeringa S, Recruiting the ABCD sample: Design considerations and procedures. Dev Cogn Neurosci. 2018;32:16–22. Epub 20180416. doi: 10.1016/j.dcn.2018.04.004. 29703560 PMC6314286

[31] World Health Organization. Public health surveillance for COVID-19: interim guidance, 2022 [updated July 22]. Available from: No. WHO/2019-nCoV/SurveillanceGuidance/2022.2. World Health Organization, 2022.

[32] Kallen MA, Lai JS, Blackwell CK, Schuchard JR, Forrest CB, Wakschlag LS, Measuring PROMIS^®^ Global Health in Early Childhood. J Pediatr Psychol. 2022;47(5):523–33. doi: 10.1093/jpepsy/jsac026. 35552435 PMC9113277

[33] Forrest CB, Bevans KB, Pratiwadi R, Moon J, Teneralli RE, Minton JM, Development of the PROMIS ^®^ pediatric global health (PGH-7) measure. Qual Life Res. 2014;23(4):1221–31. Epub 20131122. doi: 10.1007/s11136-013-0581-8. 24264804 PMC3966936

[34] Hays RD, Bjorner JB, Revicki DA, Spritzer KL, Cella D. Development of physical and mental health summary scores from the patient-reported outcomes measurement information system (PROMIS) global items. Qual Life Res. 2009;18(7):873–80. Epub 20090619. doi: 10.1007/s11136-009-9496-9. 19543809 PMC2724630

[35] Walker LS, Beck JE, Garber J, Lambert W. Children’s Somatization Inventory: psychometric properties of the revised form (CSI-24). J Pediatr Psychol. 2009;34(4):430–40. Epub 20080909. doi: 10.1093/jpepsy/jsn093. 18782857 PMC2722132

[36] Denny JC, Rutter JL, Goldstein DB, Philippakis A, Smoller JW, Jenkins G, The “All of Us” Research Program. N Engl J Med. 2019;381(7):668–76. doi: 10.1056/NEJMsr1809937. 31412182 PMC8291101

[37] Data Resourse Center for Child and Adolescent Health. The National Survey of Children’s Health 2021 [March 12, 2023]. Available from: https://www.childhealthdata.org/learn-about-the-nsch/NSCH.

[38] Bethell CD, Read D, Stein RE, Blumberg SJ, Wells N, Newacheck PW. Identifying children with special health care needs: development and evaluation of a short screening instrument. Ambul Pediatr. 2002;2(1):38–48. doi: 10.1367/1539-4409(2002)002<0038:icwshc>2.0.co;2. 11888437

[39] Read D, Bethell C, Blumberg SJ, Abreu M, Molina C. An evaluation of the linguistic and cultural validity of the Spanish language version of the children with special health care needs screener. Matern Child Health J. 2007;11(6):568–85. Epub 20070612. doi: 10.1007/s10995-007-0207-2. 17562154

[40] Centers for Disease Control and Prevention (CDC). Behavioral risk factor surveillance system 2023 [cited 2023 March 12]. Available from: https://www.cdc.gov/brfss/index.html.

[41] Sausen KA, Randolph JW, Casciato AN, Dietrich MS, Scholer SJ. The development, preliminary validation, and clinical application of the quick parenting assessment. Prev Sci. 2022;23(2):306–20. Epub 20211115. doi: 10.1007/s11121-021-01320-w. 34780005

[42] Economic Research Service USDoA. U.S. Household Food Security Survey Module 2022 [updated October 17; cited 2023 March 12]. Available from: https://www.ers.usda.gov/topics/food-nutrition-assistance/food-security-in-the-u-s/survey-tools/.

[43] RAND Corporation. Social Support Survey Instrument [cited 2023 March 12]. Available from: https://www.rand.org/health-care/surveys_tools/mos/social-support/survey-instrument.html.

[44] Freeman R, Wieling W, Axelrod FB, Benditt DG, Benarroch E, Biaggioni I, Consensus statement on the definition of orthostatic hypotension, neurally mediated syncope and the postural tachycardia syndrome. Auton Neurosci. 2011;161(1–2):46–8. Epub 20110309. doi: 10.1016/j.autneu.2011.02.004. 21393070

[45] Singer W, Sletten DM, Opfer-Gehrking TL, Brands CK, Fischer PR, Low PA. Postural tachycardia in children and adolescents: what is abnormal? J Pediatr. 2012;160(2):222–6. Epub 20111011. doi: 10.1016/j.jpeds.2011.08.054. 21996154 PMC3258321

[46] Malek S, Reinhold EJ, Pearce GS. The Beighton Score as a measure of generalised joint hypermobility. Rheumatol Int. 2021;41(10):1707–16. Epub 20210318. doi: 10.1007/s00296-021-04832-4. 33738549 PMC8390395

[47] Squires J, Bricker D, Potter L. Revision of a parent-completed development screening tool: Ages and Stages Questionnaires. J Pediatr Psychol. 1997;22(3):313–28. doi: 10.1093/jpepsy/22.3.313. 9212550

[48] Squires J BD. Ages & Stages Questionnaires^®^, Third Edition (ASQ^®^−3): A Parent-Completed Child Monitoring System. Baltimore: Paul H. Brookes Publishing Co., Inc.; 2009.

[49] Squires J, Potter L, Bricker D. The ASQ user’s guide for the Ages and Stages Questionnaires: A parent-completed, child-monitoring system Baltimore, MD: Paul H Brookes Publishing Company; 1995.

[50] Robins DL, Casagrande K, Barton M, Chen CM, Dumont-Mathieu T, Fein D. Validation of the modified checklist for Autism in toddlers, revised with follow-up (M-CHAT-R/F). Pediatrics. 2014;133(1):37–45. Epub 20131223. doi: 10.1542/peds.2013-1813. 24366990 PMC3876182

[51] Weintraub S, Dikmen SS, Heaton RK, Tulsky DS, Zelazo PD, Bauer PJ, Cognition assessment using the NIH Toolbox. Neurology. 2013;80(11 Suppl 3):S54–64. doi: 10.1212/WNL.0b013e3182872ded. 23479546 PMC3662346

[52] Squires J BD, Twombly E. Ages and Stages Questionnaire: Social-Emotional (ASQ:SE).. Baltimore, MD: Paul H. Brookes Publishing Co; ; 2002.

[53] Achenbach TM. Child behavior checklist. Burlington, VT: University of Vermont, Department of Psychiatry; 1991.

[54] Irwin DE, Gross HE, Stucky BD, Thissen D, DeWitt EM, Lai JS, Development of six PROMIS pediatrics proxy-report item banks. Health Qual Life Outcomes. 2012;10:22. Epub 20120222. doi: 10.1186/1477-7525-10-22. 22357192 PMC3312870

[55] Bevans KB, Gardner W, Pajer KA, Becker B, Carle A, Tucker CA, Psychometric Evaluation of the PROMIS^®^ Pediatric Psychological and Physical Stress Experiences Measures. J Pediatr Psychol. 2018;43(6):678–92. doi: 10.1093/jpepsy/jsy010. 29490050 PMC6005079

[56] Forrest CB, Ravens-Sieberer U, Devine J, Becker BD, Teneralli R, Moon J, Development and Evaluation of the PROMIS(^®^) Pediatric Positive Affect Item Bank, Child-Report and Parent-Proxy Editions. J Happiness Stud. 2018;19(3):699–718. Epub 20170121. doi: 10.1007/s10902-016-9843-9. 29760578 PMC5947961

[57] Chorpita BF, Yim L, Moffitt C, Umemoto LA, Francis SE. Assessment of symptoms of DSM-IV anxiety and depression in children: a revised child anxiety and depression scale. Behav Res Ther. 2000;38(8):835–55. doi: 10.1016/s0005-7967(99)00130-8. 10937431

[58] Goodman R. The Strengths and Difficulties Questionnaire: a research note. J Child Psychol Psychiatry. 1997;38(5):581–6. doi: 10.1111/j.1469-7610.1997.tb01545.x. 9255702

[59] Achenbach T, Rescorla L. Manual for the ASEBA adult forms & profiles. Burlington, VT: University of Vermont, Research Center for Children, Youth, & Families; 2003.

[60] Schrank FA, McGrew KS, Mather N. Woodcock-Johnson IV. Rolling Meadows, IL: Riverside; 2014.

[61] Adams W, Sheslow D. Wide-Range Assessment of Memory and Learning – 3rd Edition. Bloomington, MN: Pearson; 2021.

[62] Beery K, Beery N. Beery VMI: The Beery-Buktenica Developmental test of visual-motor integration with supplemental developmental tests of visual perception and motor coordination: Stepping stones age norms from birth to age six. administration, scoring, and teaching manual: PsychCorp; 2010.

[63] Science Research Associates. SRA examiner’s manual for the Purdue Pegboard. Chicago, IL: Science Research Associates; 1948.

[64] Townsend L, Kobak K, Kearney C, Milham M, Andreotti C, Escalera J, Development of three web-based computerized versions of the Kiddie Schedule for Affective Disorders and Schizophrenia Child Psychiatric Diagnostic Interview: Preliminary Validity Data Journal of American Academy of Child & Adolescent Psychiatry. 2020;59(2):309–25. 10.1016/j.jaac.2019.05.009 31108163

[65] Tasso Inc. Tasso-M20 2023 [cited 2023 March 12]. Available from: https://www.tassoinc.com/tasso-m20.

[66] Murtagh FC, P. Algorithms for hierarchical clustering: an overview. WIREs Data Mining Knowl Discov. 2011. doi: doi: 10.1002/widm.53.

[67] Monti S TP, Mesirov J, Golub T. Consensus clustering: a resampling-based method for class discovery and visualization of gene expression microarray data. Machine Learning. 2003;52(1):91–118. doi: doi: 10.1023/A:1023949509487.

